# Structure and age-dependent growth of the chicken liver together with liver fat quantification: A comparison between a dual-purpose and a broiler chicken line

**DOI:** 10.1371/journal.pone.0226903

**Published:** 2019-12-27

**Authors:** Zaher Alshamy, Kenneth C. Richardson, George Harash, Hana Hünigen, Ilen Röhe, Hafez Mohamed Hafez, Johanna Plendl, Salah Al Masri

**Affiliations:** 1 Institute of Veterinary Anatomy, Department of Veterinary Medicine, Freie Universität Berlin, Berlin, Germany; 2 College of Veterinary Medicine, School of Veterinary and Life Sciences, Murdoch University, Murdoch, Australia; 3 Institute of Animal Nutrition, Department of Veterinary Medicine, Freie Universität Berlin, Berlin, Germany; 4 Institute of Poultry Diseases, Department of Veterinary Medicine, Freie Universität Berlin, Berlin, Germany; Tokat Gaziosmanpasa University, TURKEY

## Abstract

Rearing dual-purpose chickens is a practicable approach to avoid culling one-day-old male layer chicks. The present study examined the impact of a conventional fattening diet on the liver of a novel dual-purpose chicken line (Lohmann Dual, LD) in comparison to a broiler (Ross 308) chicken line. Age-related changes of structure and lipid content of the liver were assessed. One hundred twenty and newly hatched chicks (LD = 66, Ross = 54) were kept under the same husbandry conditions and fed a commercial diet for 5 weeks for Ross and 9 weeks for LD. Six birds of each line were examined weekly. Their body weight (BW) and liver mass were recorded. Microscopic structure and ultrastructure of the liver were investigated and the liver lipid content was measured using a pre-validated method. During the study period, liver mass increased with age, while normalized liver mass decreased. Furthermore, liver mass of Ross birds was greater than that of LD birds of the same BW. Overall, no significant differences were observed in the hepatic structure or ultrastructure between the two chicken lines. The hepatic lymphatic aggregations were without fibrous capsules and their number and area increased throughout the first week, then the values began to fluctuate with age in both chicken lines. The changes in the liver lipid content of the two chicken lines were within the normal physiological range over the term of the study. The liver lipid content correlated negatively with age and body weight in both lines. It was the highest on the first day then decreased until day 7 and thereafter did not change in both chicken lines. However, given the same body weight, the Ross chickens had a 9% greater liver lipid content than LD chickens. It is concluded that there is no apparent adverse effect of a high-energy diet on the liver of LD chickens.

## Introduction

An alternative to avoid killing male hatchlings of high-performance egg-laying chickens is to use dual-purpose chickens for both meat and egg production [1]. Efforts already underway in developing such dual-purpose lines include a new hybrid chicken line, the Lohmann Dual, developed by crossing highly specialized meat and egg chicken lines [2]. The Lohmann Dual chickens have excellent growth and feed efficiency parameters when compared with other dual-purpose chicken breeds [2]. However, for further advances utilizing these intensive genetic selection processes to succeed, there is a great need for reliable data on the baseline anatomical and physiological responsiveness of the gastrointestinal tract and the liver of LD chickens when fed high-energy diets. In a recent study, we showed that LD chickens have a heavier gizzard, shorter intestine and smaller intestinal absorptive surface area than the highly specialized broiler breed, Ross 308, chickens [3]. These morphological features mirror the dynamic interface of the feed and its utilization within the gastrointestinal tract. In a similar manner, it is important to consider the role of the liver in the growth and development of highly specialized genetic lines of production birds. The intensive production conditions for rapid growth rate associated with increased metabolism have resulted in an increased work-load by the liver [4, 5].

The liver is the largest accessory gland of the avian digestive system and lies immediately caudal to the heart and lung complex. The liver is encapsulated by a thin tough capsule. Internally the liver is arranged into a series of interlinked hexagonal hepatic lobules. Each hepatic lobule has a portal canal (hepatic portal vein, proper hepatic artery, bile ductule, lymphatic vessels and vagus nerve branch) at each corner. Radially arranged linear cords of hepatocytes link the portal canals to a central vein. The basal surface of adjacent hepatocyte cords abuts elongate sinusoids that drain into the central vein. Between the hepatocytes and the sinusoidal endothelial cells are perisinusoidal spaces. The apical face of adjacent hepatic cords formed slender bile canaliculi that drain centrifugally to the nearby portal canal. A sparse collagen III fiber meshwork supports hepatocytes and sinusoids [6]. Sparse irregularly shaped lymphatic aggregations containing mainly lymphocytes are scattered throughout the liver parenchyma. In turkeys, both encapsulated and non-encapsulated lymphatic aggregations are present [7].

The liver has numerous key functions in the storage and conversion of metabolites [4]. In birds, the liver plays a major role in the synthesis and metabolism of lipids, notably because lipogenesis takes place primarily in the liver, unlike in mammals, where the adipose tissues are the site for lipogenesis [5]. *De novo* lipogenesis comprises those metabolic pathways that are involved in the synthesis of triglycerides from non-lipid precursors most commonly from dietary carbohydrate. [5]. Unfortunately, the high-energy diets used in the commercial poultry industry such as high carbohydrate diets stimulate hepatic lipogenesis [8]. Under some of these feeding regimes, pathological conditions such as the fatty liver and kidney syndrome in broilers arise [4].

This study aimed to investigate the structure of the liver in relation to age in a dual-purpose chicken line (Lohman Dual) and to compare it to that of a highly selected broiler chicken line (Ross 308). Histologically, the overall fat content of the liver was determined. The number of lymphatic aggregations and their area were measured. Ultrastructural details of the hepatocytes and sinusoids, as well as endothelial cells, hepatic stellate cells and Kupffer cells were examined using transmission electron microscopy. In addition, the area and diameters of lipid droplets were measured.

## Material and methods

### Birds and management

Two groups of eighty male chicks, the first a commercial broiler line (Ross 308) was obtained from BWE-Brüterei-Weser-Ems GmbH & Co. KG, Visbek, Germany and the second a novel dual-purpose line (Lohmann Dual, LD) was supplied by Lohmann Tierzucht, Cuxhaven, Germany. The chickens were reared in accordance with German animal welfare law. The study was approved by the Animal Welfare Committee “Landesamt für Gesundheit und Soziales”, Berlin, Germany, ID: 0236/15.

The chickens of both lines were kept under the same husbandry conditions until they reached a body weight (BW) of 2000 g, i. e. 35 days for Ross chickens and 63 days for LD chickens. The husbandry conditions and the composition of their diet were published previously [3]. The chickens had ad libitum access to a mash diet and water for the duration of the study. A starter diet (231.5 g protein and 12.6 MJ ME/kg) was fed from hatching to day 14 and then they were fed a grower diet (214.4 g protein and 13 MJ/kg) from day 15 to the end of the study. Six birds were sampled from each group in a post hatching time series: Ross = 1, 7, 14, 19, 21, 25, 28, 32 and 35 day(s), LD = 1, 7, 14, 21, 28, 32, 35, 42, 49, 56 and 63 day(s). On the sampling day, the birds were selected at random from the genetic line being examined, their live BWs were recorded, and then the birds were killed by decapitation.

### Macroscopic examination

Immediately after a bird’s death, its abdominal cavity was opened by a mid ventral abdominal incision, and the liver excised. The liver was dissected free of ligaments and associated blood vessels and weighed to an accuracy of 0.01 g on an electronic laboratory balance (AND HF-200G, Tokyo, Japan).

The normalized mass of the liver was calculated as [liver mass (g)/total BW (g)] × 100. The weekly weight gain of liver was calculated using the following relationship: (the mean liver mass in the present week − the mean liver mass in the previous week)/ the mean liver mass in the previous week) × 100.

### Specimen preparation for light microscopic examination

**Fig 1:**
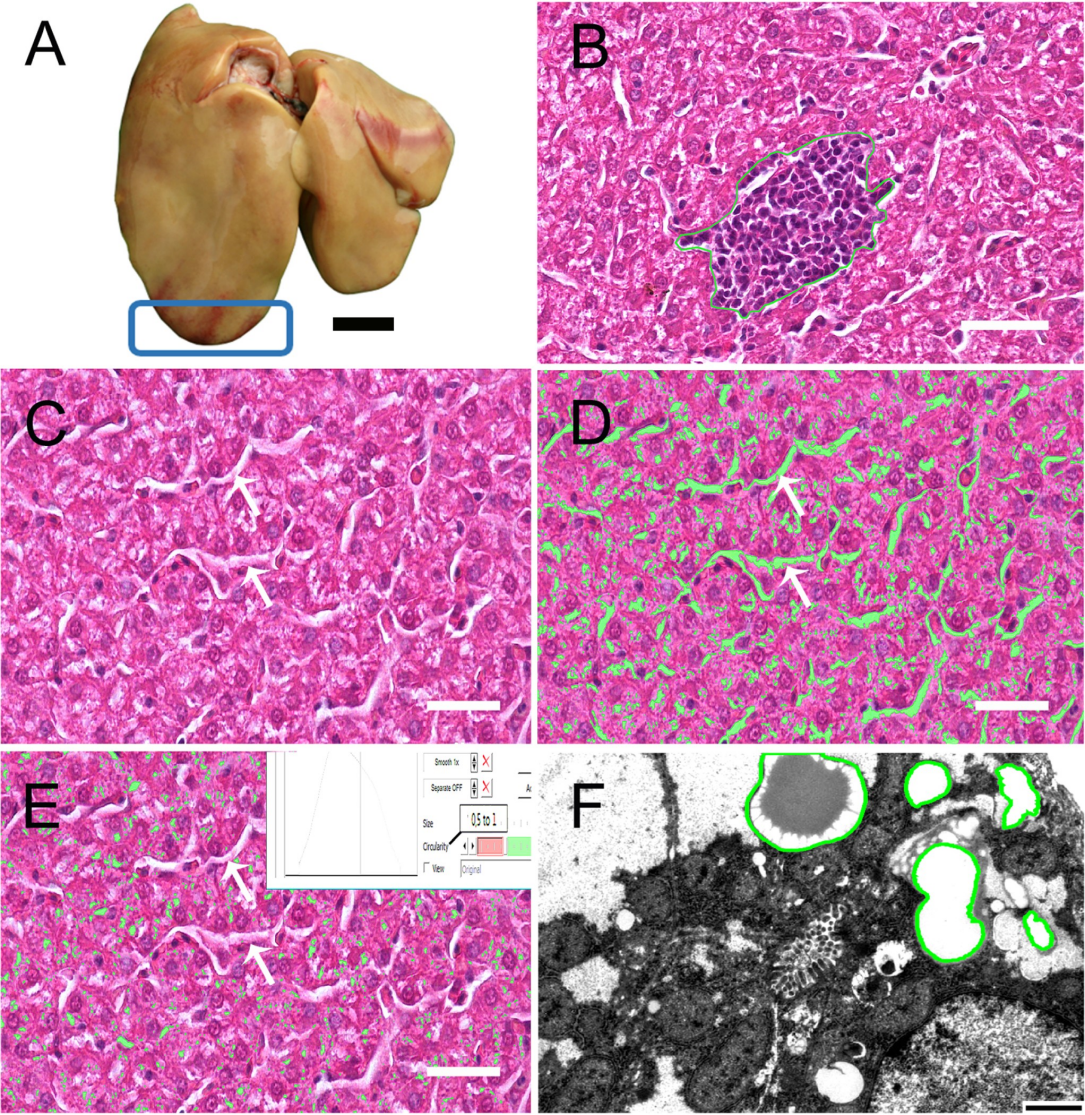
Determination of the lipid content and area and number of lymphatic aggregations in the liver. (A) parietal surface of the liver of LD chickens with the sampling site boxed for light and transmission electron microscope. (B) lymphatic aggregation outlined in green. (C) cross section of liver stained with Haematoxylin and Eosin, where arrows are sinusoids. (D) lipid content determination using a colour thresholding filter: fat accumulations and sinusoids highlighted (green) areas. (E) unspecific selection of the sinusoids (arrows) was eliminated by using shape filter with circularity degree of ≥ 50%. Bar: 1 cm for A and 25 μm for B, C, D, E, light microscopy, H&E stained. (F) liver lipid droplets outlined in green. Bar: 1000 nm for F, transmission electron microscopy.

For light microscopy, a sample (1x1x1 cm) was excised from each bird from the most caudal part of the right liver lobe (Fig 1A), and then immersed in neutral buffered formalin (4%, pH 7, 20–24°C) for 24 hours. Subsequently samples were dehydrated in an ascending graded series of ethanol, embedded in paraffin, serial sectioned at 5 μm and stained by Meyer’s Haematoxylin and Eosin (H & E).

Basic microscopic examinations of the overall liver tissue components, including lymphatic aggregations were undertaken.

Using the imaging software, NIS-Elements AR (Nikon Instruments Inc., U.S.A.), the number and the surface area of lymphatic aggregation per section per bird were estimated. At first, the surface area of each section of liver was determined and then the number of the lymphatic aggregation per 1 mm^2^ and the percentage of the surface area of the lymphatic aggregations in regard to the whole liver section was calculated. (Fig 1B).

### Lipid content measurements

#### Validation of the histological method used in this study

Here we determined the lipid content in the livers of ten 15-week-old New Hampshire × White Leghorn chickens collected, on the day of slaughter using, both chemical and histological methodologies.

For the chemical analyses the lipid content of the left liver lobe of each bird was extracted using petroleum ether according to Weende Analysis [9]. The analyzed lipid content of the liver was reported as a percentage of wet liver mass.

For the histological evaluation of lipid content, samples (1x1x1 cm) of the most caudal part of the right liver lobes were fixed in neutral buffered formalin (4%, pH 7, 20–24°C) for 24 hours. Then they were dehydrated in an ascending graded series of ethanol and embedded in paraffin wax. During the routine paraffin embedding process, lipids are removed from the liver tissue leaving behind empty circular spaces.

Serial sections were cut at 5 μm with a microtome (Jung, Histoslide, 2000 Sliding, Wetzlar, Germany). Cross-sections of liver were stained with Meyer’s Haematoxylin and Eosin (H&E) according to standard histological protocols [10].

For quantitative histological analyses of lipid content in the liver, an analyzing system (NIS-Elements AR, Nikon Instruments Inc., U.S.A.) was used. Ten fields of view (area of each field = 33333.19 μm^2^) at a magnification of 400× from around the portal canal area and distant to the large blood vessels of each sample were selected using a light microscope (Axioskop, Carl Zeiss, Jena, Germany).

Fat content was assessed by evaluating the percentage of the area occupied by lipid droplets inside the liver parenchyma. A lipid droplet was considered to be any roughly circular shaped, non-staining area (bright empty spaces) of the section (Fig 1C).

To determine the percentage of lipid droplets a chromatic followed by a morphological analysis was conducted on each field of view according to Liguori et al. (2009) [11]. In the first step a colour thresholding filter was used that highlighted all white-coloured spaces (Fig 1D) such as fat accumulations and sinusoids. In the second step a series of circular shape filters ranging from 30–70% circularity were employed to eliminate artifacts such as sinusoids.

The mean ± SD of the liver lipid content (%) derived by chemical analysis was 3.63 ± 0.79. The liver lipid content (%) estimated histologically, decreased with increasing circularity of the shape filters (Table 1). This ranged from 6.37 ± 1.01 to 2.18 ± 0.38 using 30% to 70% circularity shape filters (Table 1). To determine at which degree of circularity the closest match between the chemical and the histological analyses occurred, statistical analysis was done using Spearman's rank correlation and one-way ANOVA with Dunnett t-test for multiple comparisons. P ≤ 0.05 was considered significant. The best match between the chemical and the histological analyses was obtained with filters having a degree of circularity ≥ 50% (Fig 1E). Subsequently this filter was used to measure the lipid liver content in the LD and Ross chicken liver samples.

**Table 1:** Histological analysis of the liver lipid content using shape filters compared to the lipid content derived from chemical analysis.

Method	Lipid content (%)		Dunnett t-test	Correlation	
	Mean	SD	p-value	r	p-value
Chemical analysis*	3.63	0.79	n/a	n/a	n/a
**No shape filter**	26.47	7.27	≤ 0.001	0.19	0.603
**30% circularity filter**	6.37	1.01	≤ 0.001	0.56	0.090
**40% circularity filter**	4.91	0.79	0.001	0.65	0.043
**50% circularity filter**	3.78	0.61	0.990	0.64	0.048
**60% circularity filter**	2.90	0.47	0.093	0.62	0.054
**70% circularity filter**	2.18	0.38	≤ 0.001	0.53	0.111

n/a, not applicable; r, Pearson’s correlation coefficient; SD: standard deviation

* lipid content (%) in wet liver.

### Specimen preparation for electron microscopic examination

Samples of the most caudal part of the right lobe (0.5×0.5×0.5 cm) from two LD and two Ross birds on days 1, 7, 14, 21, 28 and 35, as well as on day 63 for the LD line were taken. The preparation of samples for electron microscopic examination was described previously [3]. Briefly, samples were fixed in Karnovsky solution, then washed in 0.1 M cacodylate buffer and incubated in 1% osmium tetroxide for 120 min. After dehydration in ethanol, the tissues were embedded in a mixture of epoxy resin, DDSA (softener), MNA (hardener) and DMP 30 (catalyst). Semi-thin sections were cut on an ultramicrotome and stained according to a modified Richardson protocol [10] for determination of the area of interest under a light microscope. The ultrathin (80 nm) sections were mounted on nickel-grids and examined with a transmission electron microscope (TEM, Zeiss EM 900; Oberkochen, Germany).

In the assessment of the TEM images, we considered a primary lysosome to be a membrane-bounded vesicle that is produced by the Golgi apparatus. Secondary lysosomes are formed when primary lysosomes fuse with phagosomes. Secondary lysosomes are larger than primary lysosomes and capable of releasing their content.

For each bird, the diameters of 40 lipid droplets as well as the number and area of the lipid droplets in 10 randomly selected fields of view (The area for each field = 244 μm^2^) (Fig 1F) were measured manually using a NIS-Elements AR analyzing system (Nikon Instruments Inc., U.S.A.). The number of lipid droplets in 1000 μm^2^ of the liver section and the area of the lipid droplets (%) were calculated.

### Statistical analysis

Data were analyzed using the statistical package program IBM SPSS Statistics 23 (IBM Corporation, New York, USA). The graphs presented were made by using the statistical package program JMP® Pro 13 (SAS Institute Inc., Cary, USA). Comparisons of the two lines of the same age groups were performed using the Mann–Whitney U test. The relationship between the liver mass or lipid liver content and age was assessed using one-way analysis of variance (ANOVA) with the post hoc test Least Significant Difference (LSD). To explore the effect of chicken line and BW on the liver mass and lipid liver content, all data collected were regressed against the genetic line and BW using the log-log regression model. To test the relation between fat deposition in the liver and the body weight as well as the age, Pearson's correlation coefficient was used. A p value of < 0.05 was considered significant.

## Results

### Gross anatomy

Once the abdominal cavity was opened and the sternum and ribs were elevated, the liver was visible lying ventrally in the viscus. The liver’s colour varied depending on the bird’s age. The liver was pale-brown on the day of hatching due to the abundant yolk pigment but by the end of the first week, it darkened to a light brown. Thereafter, the colour ranged from light brown to reddish brown.

**Fig 2:**
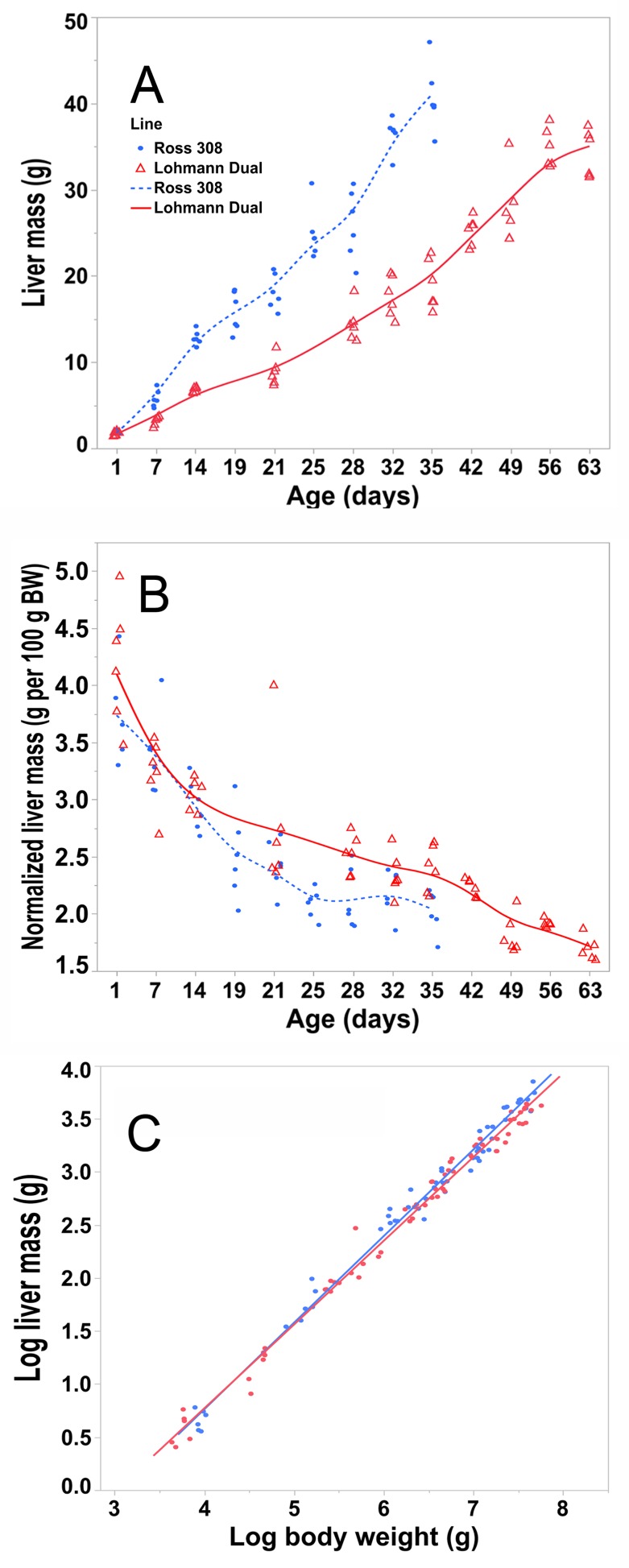
Liver mass and normalised mass of both chicken lines. Trendlines of the changes in mass (A) and normalised mass (B) of livers versus days post hatching for Ross and LD chicken lines. (C) linear regression line of logarithm of liver mass versus logarithm of total body weight for both chicken lines. Symbols represent each individual value for each chicken line. BW, body weight.

The liver mass of the LD chickens increased by 0.51 g/day (d) from d 1 to 35 post-hatching, whereas the liver of Ross chickens increased by 1.14 g/d over the same period. From d 1 to 63 the LD liver mass increased by a rate 0.52 g/d. The liver grew gradually with age in both lines (Fig 2A), with the mass of the liver increasing by 19.1 times in LD birds and 20.87 times in Ross birds by the end of the study. The greatest weight increase of the liver (196.66% for Ross and 107.73% for LD) was over the first week of life for Ross and over the second week for LD chickens. After day one, the liver masses of LD chickens were lighter than those of Ross chickens (p < 0.01). On the first day of life the normalized liver mass peaked at 4.2 ± 0.53% for LD and 3.7 ± 0.4% for Ross chickens, after which there was a gradual decrease in the contribution of the liver to overall body weight (Fig 2B). The liver of LD chickens contributed a greater percentage of total body weight on days 28, 35 than that of Ross chickens (p < 0.05).

According to regression analysis, both body weight and chicken line had an influence on the liver mass, p ≤ 0.01, adjusted R^2^ = 0.99. The liver mass of Ross birds was heavier on average by 2.3% than that of LD birds of the same BW (Fig 2C).

### Microscopic examination of the liver

#### Light microscope

The overall histological structure and appearance of the liver of both genetic lines was similar. No pathological changes were observed in the chickens’ livers.

**Fig 3:**
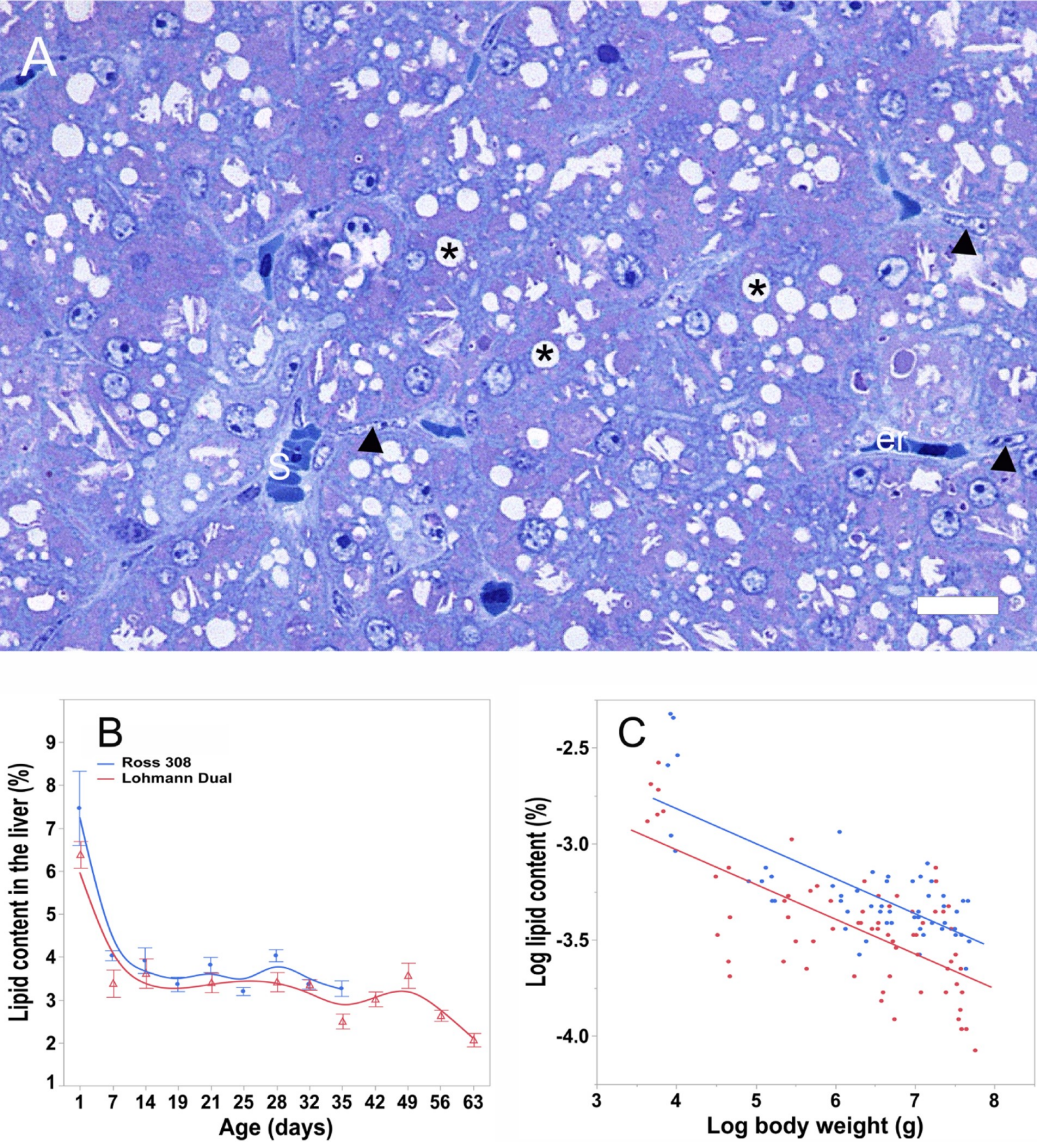
Liver lipid content (%) estimated via histological method. (A) Semi-thin section of a 1-day-old LD chick liver stained with Richardson blue. Where, (er) erythrocyte, (s) sinusoid, (asterisks) lipid droplets, (arrow) endothelial cell. Bar: 10 μm. (B) lipid liver content % versus age, bars refer to mean ± standard error of the mean of the sampled chicken at each time interval. (C) linear regression line of liver lipid content versus body weight. Symbols represent each individual value for each chicken line.

The liver surface was covered by a thin loose connective tissue layer. Below this layer was a thin capsule of dense connective tissue that extended into the liver lobes and divided the liver into lobules. However, interlobular connective tissue was scant and difficult to distinguish. The hepatocytes were grouped into hepatic lobules. At the periphery of each lobule portal canals were found that consisted of the interlobular branch of the hepatic artery, interlobular branch of the portal vein, bile ductules as well as less visible lymphatic vessels and nerve branches. In the centre of each lobule, a large central vein was present. The hepatic parenchyma primarily consisted of rows of conically shaped hepatocytes flanking elongate sinusoids (Fig 3A). The hepatocytes were attached to each other in a hexagonal arrangement forming hepatic plates. The hepatic plates were arranged irregularly inward from the periphery edge of each liver lobe towards the central vein.

Lymphatic cells in the liver were formed into aggregations that varied in number (0.17 to 4.5 per mm^2^) and in area (0.41 to 3.42% of the histological section).

In both chicken lines these aggregations primarily had a heterogenous distribution throughout the liver parenchyma but some aggregations were associated with portal canals. The aggregations characteristically consisted of a solitary population of tightly packed small lymphocytes. The lymphocytes were characterized by large, dark, round nuclei, and very little cytoplasm. These aggregations did not have capsules, sinuses, cortices, medullae, or germinal centers. Their peripheral borders were irregularly shaped and varied considerably in size (Fig 1B).

The absolute number and area of the lymphatic aggregations per 1 mm^2^ of histological section are presented in Table 2. These values varied in the different age groups. The number and area of lymphatic aggregations increased sharply over the first week of life in both lines. After day 7, the number and area of the lymphatic aggregations varied without any discernable pattern.

**Table 2:** Area and number of the lymphatic aggregations in histological sections of LD and Ross chickens.

Age (days)	Line*	Lymphatic aggregation area %		Number of lymphatic aggregations in mm^2^	
		Mean	SD	Mean	SD
**1**	**LD**	0.18	0.28	0.50	0.55
	**Ross 308**	0.41	0.93	0.17	0.41
**7**	**LD**	0.99	0.56	4.50	1.64
	**Ross 308**	1.02	0.58	3.33	1.86
**14**	**LD**	0.90	0.54	4.50	2.51
	**Ross 308**	0.74	0.45	3.17	1.33
**19**	**Ross 308**	1.01	0.83	3.00	2.00
**21**	**LD**	0.83	0.46	2.50	1.05
	**Ross 308**	0.63	0.54	2.00	1.10
**25**	**Ross 308**	2.37	1.77	3.83	1.83
**28**	**LD**	1.02	1.62	2.00	1.67
	**Ross 308**	0.43	0.35	0.83	0.75
**32**	**LD**	1.75	1.97	2.67	0.52
	**Ross 308**	1.42	1.12	3.00	1.67
**35**	**LD**	1.12	0.95	1.83	0.98
	**Ross 308**	1.82	1.17	2.67	1.63
**42**	**LD**	3.42	4.19	2.67	1.97
**49**	**LD**	1.19	0.90	2.33	1.75
**56**	**LD**	1.48	0.79	4.17	3.13
**63**	**LD**	1.32	0.72	3.67	1.75

LD: Lohmann Dual; Ross: Ross 308; SD: standard deviation; %, percentage of the lymphatic aggregations in the field of view area.

*6 birds in each group for each chicken line.

#### Liver lipid content in LD and Ross chickens

The percentage of lipid in the liver of both lines correlated negatively with age as well as with body weight (r = -0.56 for LD chickens and r = -0.52 for Ross chickens, p ≤ 0.001 for body weight; r = -0.97 for LD chickens and r = -0.96 for Ross chickens, p ≤ 0.001 for age).

The lipid percentage was highest in one-day-old chickens in both lines (Table 3). From day 7 until day 35 for Ross chickens and until day 49 for LD chickens, there were little differences between the age groups. The lipid percentage in LD chickens decreased from day 49 to the end of the study (Fig 3B).

**Table 3:** Percentage of lipid storage in the liver of LD and Ross chickens assessed by light microscopy.

Age (days)	Line*			
	Lohmann Dual (%)		Ross 308 (%)	
	Mean	SEM	Mean	SEM
**1**	6.4	0.3	7.5	0.9
**7**	3.4	0.3	4.0	0.1
**14**	3.6	0.3	3.9	0.3
**19**	n/a	n/a	3.4	0.2
**21**	3.4	0.2	3.8	0.2
**25**	n/a	n/a	3.2	0.1
**28**	3.4	0.2	4.0	0.1
**32**	3.4	0.1	3.4	0.1
**35**	2.5	0.2	3.3	0.2
**42**	3.0	0.2	n/a	n/a
**49**	3.6	0.3	n/a	n/a
**56**	2.6	0.1	n/a	n/a
**63**	2.1	0.2	n/a	n/a

LD: Lohmann Dual; Ross: Ross 308; SEM: standard error of the mean; n/a: not applicable, %, percentage of the lipid areas in the field of view area.

*6 birds in each group for each chicken line.

According to regression analysis, both BW and chicken line, had an influence on the lipid percentage, p ≤ 0.001, adjusted R^2^ = 0.54. The lipid percentage in the liver of Ross chickens was greater on average by 9.1% than that of LD chickens of the same BW (Fig 3C).

#### Electron microscopy

**Fig 4:**
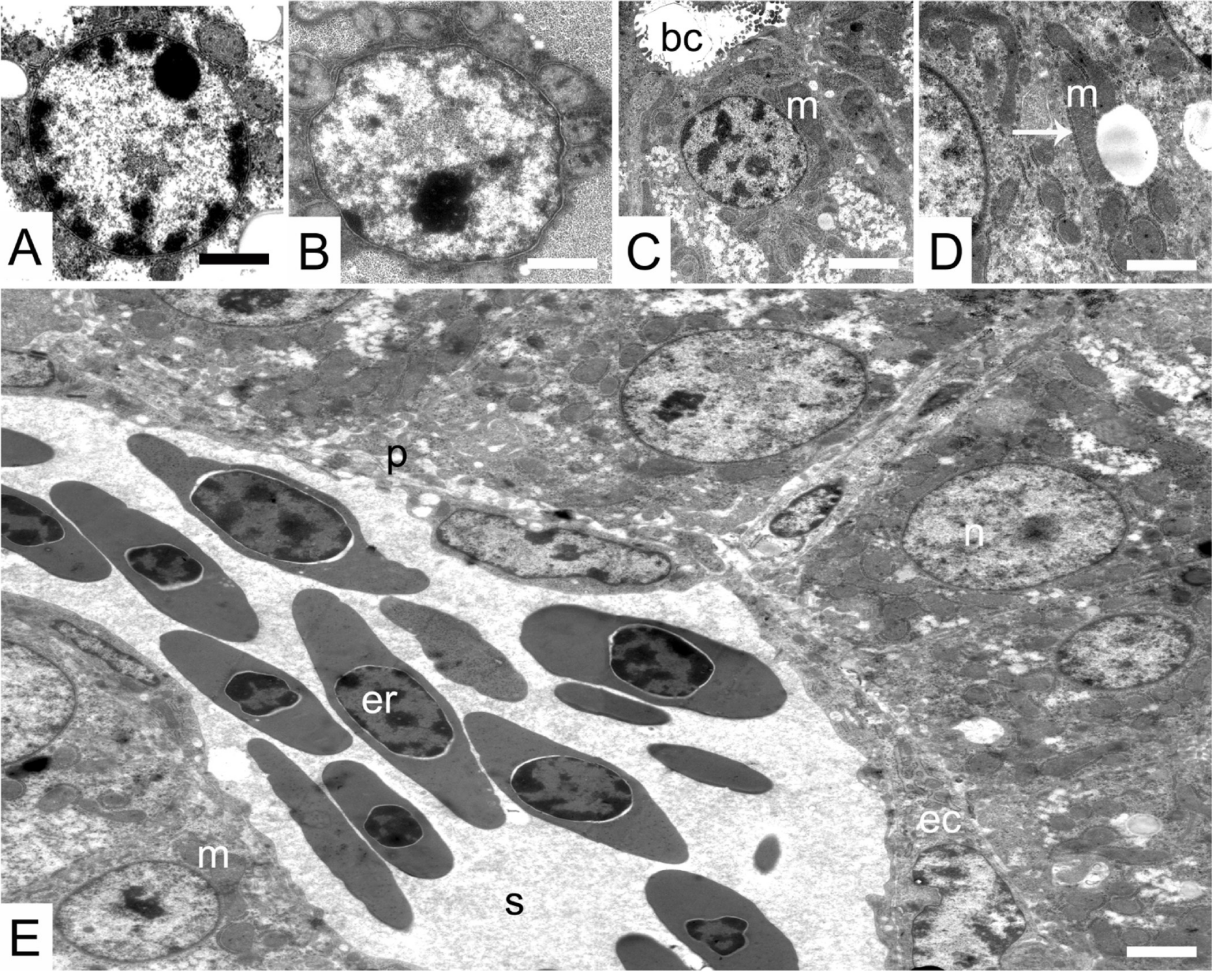
Transmission electron micrographs of the liver of 1 and 7 day old chicken of both lines. (A and B) hepatocyte nuclei of 1-day-old Ross (A) and LD chicks (B). (C and D) concentrations of mitochondria in 7-day-old LD (C) and Ross chicks (D). (E) a panorama micrograph of hepatocytes and sinusoid of 7-day-old LD chicks. Where, (bc) bile canaliculus, (ec) endothelial cells, (er) erythrocyte, (m) mitochondria, (n) nucleus, (s) sinusoid lumen. (p) perisinusoidal space. Symbols: (arrow) mitochondria associated membranes. Bar 1000 μm.

The overall appearance and structure of the liver of LD chickens were similar to that observed in Ross chickens. The hepatocytes of LD and Ross chickens were conical in shape and their spherically shaped nuclei were located basally adjacent the sinusoid. On day 1, the hepatocytes of Ross chicks appeared to have the dense heterochromatin (Fig 4A) in the peripheral region of the nucleus, more densely packed than that of LD chicks (Fig 4B). By day 7, the heterochromatin was less densely packed than that observed on day 1 in both chicken lines. The nucleolus was irregular in shape in both lines.

The hepatocytes were arranged radially around the bile canaliculi. Their narrow apical poles had short finger-like projections (microvilli) that protruded into the lumina of the adjacent bile canaliculi (Fig 4C). Tight junctions were seen between adjacent hepatocytes near the bile canaliculi. The wide basal poles of the hepatocytes were oriented towards the liver sinusoids. They had microvilli that projected into the perisinusoidal space (Fig 4E).

**Fig 5:**
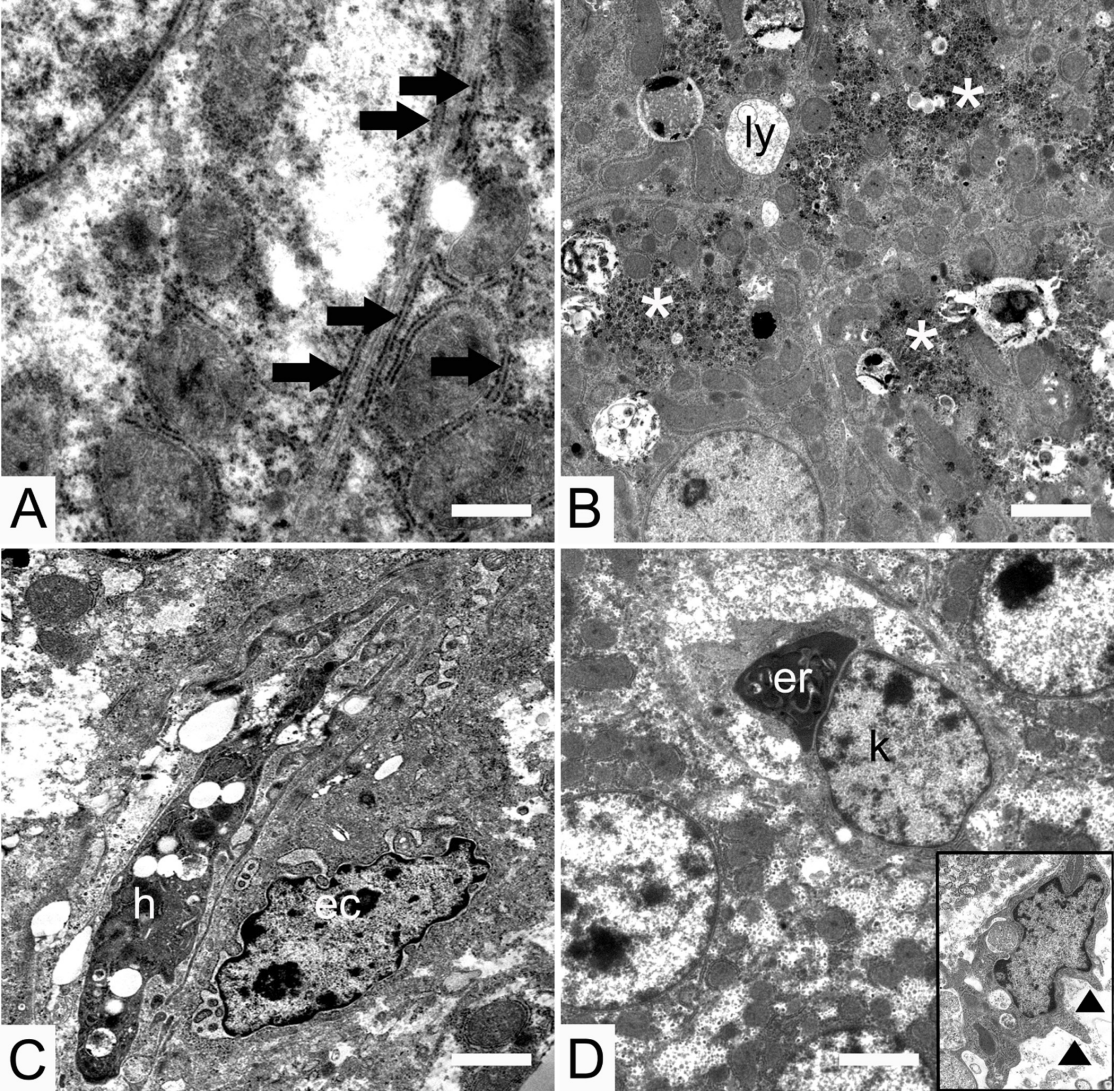
Transmission electron micrographs of the livers of both chicken lines. (A) neighbouring hepatocytes in a 7-day-old LD chick, where the rough endoplasmic reticulum is arranged beneath the plasma membrane (broad arrows), mitochondria associated membranes (thin arrows). (B) glycogen accumulations in the liver of 14-day-old Ross chicks where (ly) lysosomes, (asterisk) glycogen accumulations. (C) hepatic stellate cell (Ito cell) (h) of a 1-day-old LD chick with a neighbouring (ec) endothelial cell. (D) erythrophagocytosis by Kupffer cells in a 1 day Ross chick where (er) erythrocyte, (k) nucleus of Kupffer cell, (arrowhead) pseudopod. Bar 1000 nm.

In both chicken lines the hepatocytes’ cytoplasmic organelles were less numerous and densely packed in one-day-old chicks than in seven-day-old chicks (Fig 4B and 4D). The hepatocyte mitochondria were scattered throughout the cytoplasm with the greatest concentrations being perinuclear (Fig 4B). The mitochondria varied greatly in size and ranged from oval to rod-shape. Ovoid mitochondria were dominant in day old chickens of both genetic lines (Fig 4A and 4B). The rod mitochondria were first seen on day 7 (Fig 4C and 4D). From day 7 onwards there were greater concentrations of mitochondria consisting equally of ovoid and rod forms (Fig 4C and 4D). The mitochondria were associated closely with the endoplasmic reticulum, forming mitochondria-associated endoplasmic reticulum membranes (MAM) (Fig 4D). Here, the endoplasmic reticulum was aligned parallel to the mitochondrion periphery. The rough endoplasmic reticulum (RER) of the hepatocytes appeared abundant in the cytoplasm. It was arranged also beneath the plasma membrane (Fig 5A).

Lysosomes were distributed randomly within the cytoplasm and were variable in size and in the nature of their internal content (Fig 5B). Lysosomes were seen in their primary state as vesicles without electron dense content and in their secondary state as vesicles with electron dense content. The largest number of lysosomes occurred on day 14 (Fig 5B).

Glycogen accumulations were seen as dark spots scattered throughout the cytoplasm. In both chicken lines the largest accumulations of glycogen were seen on day 14 (Fig 5B).

Within the hepatocytes lipid droplets were seen to be concentrated near the bile canaliculi. The droplets were spherical in shape and showed variation in electron density in both lines (Fig 1F). Lipid droplets were observed in different sizes and did not have a membrane (Fig 1F). Some small droplets fused together forming larger droplets (Fig 1F). The diameter and area of the lipid droplets of the liver were similar in both lines (Table 4). In both chicken lines the number of the lipid droplets peaked on the first day of life and then decreased steadily such that by day 14 only about 50% remaine (Table 4). From day 14 onward, the number of the lipid droplets did not differ greatly (Table 4). The greatest, percentage of liver fat and diameter of lipid droplets were observed on the first day of life in both lines (Table 4). By day 7, the fat percentage and droplet diameter had decreased sharply. After day 14 the changes in the fat percentage and diameter were slight in both chicken lines (Table 4).

**Table 4:** Lipid droplets percentage, number and diameter in the liver of LD and Ross chickens versus days post hatching under TEM.

Age	Line	Lipid content (%)		Lipid droplets number in 1000 μm^2^ of the liver surface		Lipid droplets diameter (μm)	
		Mean	SD	Mean	SD	Mean	SD
**1**	**LD**	12.26	7.91	43.42	40.58	3.93	1.34
	**Ross**	18.71	8.44	33.31	15.25	4.67	1.10
**7**	**LD**	1.47	1.43	31.16	30.67	0.83	0.22
	**Ross**	4.34	3.87	27.65	16.44	1.30	0.27
**14**	**LD**	1.23	1.34	15.11	7.15	1.54	0.81
	**Ross**	2.31	1.77	17.35	11.07	1.57	0.61
**21**	**LD**	1.62	1.11	12.25	7.13	1.31	0.45
	**Ross**	0.46	0.56	11.23	4.93	1.27	0.78
**28**	**LD**	2.33	1.37	18.78	6.68	1.16	0.37
	**Ross**	1.61	1.17	13.06	6.16	1.24	0.45
**35**	**LD**	1.84	1.43	20.25	14.69	1.07	0.36
	**Ross**	1.93	1.22	34.86	23.79	1.03	0.29
**63**	**LD**	1.32	0.88	26.54	12.39	0.91	0.44

LD: Lohmann Dual; Ross: Ross 308; SD: standard deviation; %, percentage of the lipid to the field of view area.

The walls of the liver sinusoids were lined by a single layer of endothelial cells. The endothelial cells were elongate in shape and had an elongate oval nucleus. The endothelial cell cytoplasm was rich in organelles.

Hepatic stellate cells (Ito cells) were localized in the perisinusoidal space. They were irregular in shape (between oval to more or less elongated), had oval nuclei and were rich in endoplasmic reticulum (Fig 5C). They were smaller in size and had fewer mitochondria than did the hepatocytes. In both chicken lines the hepatic stellate cells were seen to be associated frequently with the nuclear region of endothelial cells (Fig 5C). The lipid droplets in hepatic stellate cells appeared to be smaller than those seen in the hepatocyte cytoplasm.

Stellate macrophages (Kupffer cells) were observed in the sinusoidal lumen, in close contact with the endothelial cells. The shape of Kupffer cells varied from spherical to spindle-shaped and they had large triangular to oval-shaped nuclei. Their cytoplasmic membrane possessed conspicuous pseudopodia that protruded into the sinusoidal lumen (Fig 5D). Erythrophagocytosis by Kupffer cells was observed (Fig 5D).

## Discussion

The essential requirement for rapid growth in young chickens is the rapid development of their digestive tract and liver relative to their pectoral muscle mass [12]. Scheele (1997) reported that during the early stages of development, growth is most marked in the alimentary tract and liver whilst that of the pectoral muscle mass lags behind. However, in the later stages of development, massive increases in pectoral muscle mass coupled with feather growth dominates [13]. Both of these are metabolically demanding and are based on the effective efficiency of the digestive system. In our previous paper [3] we reported that the length and mass of the gastrointestinal tract in relation to body weight were highest throughout the first week of life in both LD and Ross chickens. In the current study we found that the proportional mass of the liver was highest on the first day of life in both chicken lines.

As would be expected, the peak normalized liver mass found in one-day-old chickens of both lines corresponds to the necessary essential liver capacity required for their subsequent development. When limited hepatic capacity occurs, it has large-scale consequences such as suboptimal growth as well as non-specific clinical symptoms [14, 15]. After day one the liver growth in both LD and Ross chickens followed a similar pattern of slow decrease. This is in agreement with the previous findings of a reduction in the relative liver mass of broilers with age [16–18].

The microscopic structure of the liver of both chicken lines was similar to that reported previously [6, 19]. In chickens, there were no significant collagen septae between hepatic lobules [20]. This may explain why the connective tissue between liver lobules of LD and Ross chickens was difficult to distinguish.

The scattered lymphatic aggregations in both chicken lines were a normal feature of their liver parenchyma. Olah et al. (2014) reported that lymphoid aggregations develop in non-lymphoid organs such as the liver, pancreas, kidney, endocrine glands, gonads, brain and spinal cord [21]. Hünigen et al. (2016) reported that non-encapsulated lymphatic aggregations were more common than encapsulated lymphatic aggregations in the liver of the turkey [7]. However, in this study all lymphatic aggregations were non-encapsulated. In general, diffuse lymphatic aggregations are sites where the lymphocytes come into contact with antigens that subsequently stimulate ongoing proliferation of lymphocytes and promote B lymphocytes to become plasma cells, that produce antibodies [22]. This is supported by Younus et al. (2017) who reported that broiler chickens affected by viral hepatitis had diffuse inflammatory foci composed primarily of lymphocytes and macrophages in their liver parenchyma [23]. Increased periportal infiltrates of mixed lymphocytes and phagocytic cells were also observed in the avian liver affected by Borrelia bacteria [24].

The hepatic ultrastructure of LD and Ross chickens is comparable to previous observations of birds’ liver [6, 25–27]. In our study only mononucleated hepatocytes were observed in both chicken lines. This contrasts to mammals where binuclear hepatocytes predominate [28]. The hepatocytes of both LD and Ross chickens had abundant, closely packed, large mitochondria confirming similar observations reported by Tanuma and Ito (1978) who concluded that the abundant large mitochondria in bat hepatocytes were necessary for the production of the large amount of energy required for flying [29].

Historically the standard semi-quantitative evaluation of lipid droplets in the liver was based on a grading scale from 0 to 3 and expressed as a percentage where 0 is < 5%; 1 is 5%–33%; 2 is 34%–66%; and 3 is > 66%) [30]. However this protocol has been claimed to “overestimate” the fat deposition [31] as well as to be “inaccurate and subjective” [11]. Liguori et al. (2009) applied automated image analysis software using chromatic and shape filters to examine the liver fat content on histological sections in rats. They found that using only a colour filter to select areas of lipid accumulation overestimated the percentage fat content due to the automatic inclusion of sinusoids that had the same colour profile. They circumvented this problem by using a shape filter that excluded the sinusoids [11]. Our study improved upon earlier studies because it used both colour and circularity filters and validated these results against those derived from more accurate chemical analyses of the same samples. However, 2D measurements may do not mirror the actual parameters and the distribution of lipid droplets in the liver may vary according to the sampling locations. Moreover, maintaining a constant cite of investigation may introduce a constant error. Therefore, the significance of sampling should be considered when analysing tissues. Standard sampling designs and procedures are available for stereological and histological tissue analysis for various organs and species [32]. In the literature, there is no preferred or specific sampling site from the liver in chicken. However, a recent study by Gerspach et al., (2017) reported that the lipid distribution in 10 different locations of the liver in 30 cows had only minor variation in the distribution of lipids at the histological level [33].

To overcome these issues, the same procedure to assess the liver lipid content, which we validated previously, was applied on both chicken lines; Ross 308 and Lohmann Dual. We believe that maintaining a constant sampling site makes our data reliable to compare the liver lipid content between Ross 308 and Lohmann Dual chickens.

The lipid content curves of each chicken line were nearly identical over the study period whether measured using images from light microscope or TEM. Our finding that the percentage of lipid in the liver of both lines is correlated negatively with age and body weight is in line with previous results [34]. In both chicken lines the liver lipid content was highest on the day of hatching in both histological and ultrastructural sections. Over the next few days post-hatching, the chicks were dependent on yolk lipid as they transitioned to their independently obtaining adequate nutrients and energy from their oral feed intake. Noble and Cocchi (1990) reported that during the incubation period of the chick, 80% of the yolk lipid content is mobilised and absorbed by embryonic tissues [35]. Then over several days post-hatching the chicks utilised the remaining yolk lipid as their main energy source. The decrease in the liver lipid content over the first week corresponds to their consuming the yolk lipid and their eating their starter formula [35]. Our study supports this by our noting that the normalized liver mass was greatest over the first week. This suggests that the liver is relatively functionally mature at hatching and that the final functional maturation is rapid as the chickens’ transition from fat-rich yolk stores to a predominantly carbohydrate diet [18]. For the remainder of their lives the liver lipid content in both chicken lines ranged between 2.1–4.0%. This is in agreeance with previous values for total liver lipid percentages ranging between 1.6–3.2% of the wet weight of the liver from day 22 to 36 [36]. The changes in liver lipid content from the early days post hatching and onwards are attributed to the changing role of the liver from being mainly a depository organ during the first days post hatching to one of synthesizing fat for both structural and functional purpose afterwards [34].

A strain or breed of chickens is, more or less, sensitive to dietary or hormonal effects on hepatic lipid accumulation [37]. Genotype is one of the main factors affecting fat deposition in the broiler chickens in addition to sex, age and the nutrition. The origin of fats in a broiler chicken’s body is by means of exogenous (from the diet) or endogenous (synthesized in the liver) fat [38]. This suggests that Ross chickens could be genetically more prone to deposit fat in the liver than LD chickens of the same body weight. Commercially, feeding chickens high-energy diets based on a high percentage of carbohydrate stimulates fat deposition in the liver [8]. In addition to this, the rapid growth pattern of modern broiler chicken lines has been accompanied by an increase in voluntary feed intake that has led to increased fat deposition in the body [39]. This is a partial explanation of the higher liver lipid content of Ross chickens than that of LD chickens, in birds of the same body weight.

In our study, as there were no pathological changes in the structure and ultrastructure of the liver and no significant difference in the lipid content of the liver in the comparable age groups of Ross and LD chickens, it can be assumed that the liver lipid content of both genetic lines is within the normal physiological range. Our results suggest that the livers of LD chickens were able to metabolise high-energy diet used without side effects.
